# Are We on the Way to Successfully Educating Future Citizens?—A Spotlight on Critical Thinking Skills and Beliefs about the Nature of Science among Pre-Service Biology Teachers in Germany

**DOI:** 10.3390/bs13030279

**Published:** 2023-03-22

**Authors:** Virginia Deborah Elaine Welter, Lars Emmerichs-Knapp, Moritz Krell

**Affiliations:** 1 IPN—Leibniz Institute for Science and Mathematics Education, 24118 Kiel, Germany; 2 Institute of Biology Education, University of Cologne, 50931 Köln, Germany

**Keywords:** critical thinking, nature of science, 21st century skills, pre-service teachers, teacher education, learning opportunities

## Abstract

A rapidly changing world and constantly expanding knowledge requires education to no longer focus on teaching subject-matter knowledge but also to promote students’ critical thinking (CT) and an accurate understanding of the nature of science (NOS). However, several studies have shown that these skills are still poorly acquired during formal education. Given the cause–effect sequence from teacher education to teacher action to student learning, it seems reasonable to consider individual factors on the part of (pre-service) teachers as possible contributors to such skill gaps. In our study, we therefore investigated how pre-service biology teachers perform on tasks assessing their CT skills and NOS beliefs. In addition, we addressed the questions of whether test performance and/or the relationships between CT skills and NOS beliefs differ as a function of the number of learning opportunities. Our results show that our participants’ CT skills were only in the low–average range. Moreover, 86% of them did not have an informed understanding of NOS. Although participants in the master’s program demonstrated clearly superior CT skills than those in the bachelor’s program, no such difference was found in terms of NOS beliefs. However, there was a consistent advantage for pre-service teachers who were aspiring to a teaching qualification in two (as opposed to only one) scientific subjects. Our findings provide useful implications, particularly with respect to the influence of learning opportunities in university teacher education and the effectiveness of CT- and NOS-based instructional settings. On a more prospective note, our findings underscore that, given the grand global tasks of the 21st century, it seems more important than ever to ensure that pre-service science teachers have sufficient expertise in CT and NOS in order to increase the likelihood that these teachers will be able to successfully help their future students develop these skills.

## 1. Introduction

Socio-scientific issues (SSI) are complex and controversial topics that involve scientific concepts and have significant social, ethical, or moral implications [1]. In recent years, SSI have become increasingly important. These issues range from global concerns such as climate change, genetic engineering, health issues, and food safety to local problems such as the use of pesticides in agriculture or water pollution. Thus, SSI have a significant impact on individuals, communities, and the planet and require interdisciplinary approaches and solutions [1].

The role of science in addressing SSI is crucial, as science provides the empirical evidence and knowledge needed to understand these complex issues [2]. Biology in particular plays a key role, considering that many of the most pressing current and future SSI are fundamentally biological in nature. As the study of living organisms and their interactions with the environment, biology can contribute to a deeper understanding of issues such as human health, biodiversity, or ecosystem sustainability [3].

However, addressing SSI requires more than just scientific knowledge. It also requires critical thinking (CT) skills, an understanding of the nature of science (NOS), and the ability to integrate different perspectives and values [1]. Thus, for science education in general and biology education in particular, SSI raise important questions about the best way to enable learners to acquire knowledge in a reflective and self-directed way, to understand and explain their environment, and to elaborate rationally and critically on (current and future) SSI [4]. However, in view of a rapidly changing world, constantly expanding knowledge, and the limited time available for formal education, it is necessary to identify priorities to be set in any curriculum [5]. In this regard, the international discourse on science education has increasingly claimed that school and university education should no longer primarily focus on the acquisition of subject-matter knowledge but should place more emphasis on the teaching of rather generic skills (for an overview, see [6]). This means that, in addition to teaching specialist knowledge (e.g., stages of meiosis or the chemical structure of methane), students’ scientific reasoning (SR) skills, including an accurate NOS understanding, should be promoted [7,8,9]. Thus, the focus is increasingly shifted from predominantly asking “What?” (declarative knowledge) to the questions of “How?” (procedural knowledge) and “Why?” (epistemic knowledge) [8,10,11]. Such a deeper understanding of scientific concepts is associated with improved knowledge transfer and generalization [7,12,13], which is important for constructive engagement with SSI. Moreover, considering the exponentially increasing body of scientific knowledge and, at the same time, the general availability of any kind of (mis-)information via the internet, it enables students to critically evaluate scientific information in order to make evidence-based decisions [5]. Such critical judgement addresses learners’ CT, which is defined as the ability “to carefully evaluate and judge statements, ideas and theories relative to alternative explanations” [14] (p. 23). Scientific subjects therefore provide an excellent framework for promoting CT as it shares many similarities with SR [8,15,16]. Accordingly, Morris et al. defined SR as “the reasoning and problem-solving skills involved in generating, testing and revising hypotheses or theories, […] reflecting on the process of knowledge acquisition and knowledge change that results from such inquiry activities” [17] (p. 61). Complementing this, scientific subjects’ methodological and epistemological lines of reasoning provide a framework for reflection that goes beyond individual disciplines such as biology, chemistry, or physics [18].

Because SR and related constructs such as CT and NOS are such essential learning goals in science education [19,20], many recent reform documents worldwide have emphasized them (more) explicitly—for example, the Next Generation Science Standards (NGSS) [21], the Australian STEM School Education Strategy 2016–2026 [22], and the national curricula in England [23], France [24], and Taiwan [25]. Despite such reforms, however, there is still some need for further improvements. In 2019, Lederman et al. conducted a systematic assessment of the understanding of scientific inquiry (SI) of 2634 seventh graders in 18 different countries on six different continents. For this purpose, they used a measurement instrument [26] based on a definition of understanding of SI that included both declarative and procedural knowledge and, in particular, central aspects of NOS and CT. Their findings indicate that the students’ overall understanding of SI is very poor. Furthermore, the authors identified some common aspects in the education systems of the countries studied that might have contributed to this result: insufficient embedding in educational standards, and non-reflective, action-oriented learning, accompanied by a lack of explicit teaching on understanding SI [20].

Lederman et al.’s results were also found for German students [20]. Considering the cause–effect sequence from teacher education to teacher action to students’ learning success [27], it seems reasonable to also consider individual factors on the part of (pre-service) teachers as possible co-causes of students’ competency gaps. A similar conclusion was also reached by Tommasi et al. [28] in their systematic review on the promotion of CT skills and media literacy in the context of initial vocational education. Accordingly, the authors advocated for specific training to help teachers develop these skills. Against this background, our study explored how pre-service teachers from Germany perform on tasks assessing their CT skills and NOS beliefs. In addition, we addressed the questions of whether test performance and/or the relationships between CT skills and NOS beliefs differ as a function of the number of learning opportunities.

### 1.1. Critical Thinking

As early as 1910, John Dewey mentioned CT as an overall educational goal, which he called a “scientific attitude of mind” [29] (p. iii). Especially in recent years, a renaissance of this call can be observed. In view of students’ autonomy and their preparation for an empowered life and active citizenship, CT has been identified as a key 21st century skill [6]. However, there are numerous definitions of CT, many of which focus on the same basic concept of careful and goal-directed thinking, but differ in terms of the criteria for carefulness, the nature of the goal, or the scope and elements of such thinking [30]. One of the most widely cited definitions refers to a Delphi study [31] led by Peter Facione in 1990 that involved 46 experts who developed a consensus statement regarding the prototypical critical thinker. According to this study, CT has been defined as “purposeful, self-regulatory judgment which results in interpretation, analysis, evaluation, and inference, as well as explanation of the evidential, conceptual, methodological, criteriological, or contextual considerations upon which that judgment is based” [31] (p. 3). In addition, the experts identified cognitive skills and affective dispositions that generally characterize critical thinkers. In this regard, observable CT is a direct manifestation of the underlying cognitive skills, whereas the dispositions are facilitating or inhibiting in applying the skills [32]. In total, six key CT skills were identified: (1) interpretation, (2) analysis, (3) evaluation, (4) inference, (5) explanation, and (6) self-regulation [31]. Important dispositions are, for example, inquisitiveness, willingness to inquire, open-mindedness, honesty in facing one’s own biases, or reasonableness in selecting and applying criteria [31]. Conversely, this means that most dispositions that are barriers to CT (e.g., a preference for order and predictability, discomfort with ambiguity, or closed-mindedness) can be associated with a construct known as the need for cognitive closure (NCC) [33,34,35,36,37,38].

To date, however, controversy exists regarding the question of the extent to which CT is a domain-specific or a generic skill [30,39]. Nevertheless, there is agreement on two points: (1) There are several generic CT principles, such as the hypothetico-deductive method, systematicity, and evidence-orientation, but (2) the application of these generic principles in a particular situation always requires specific background knowledge in the domain to which that situation belongs [30,31,40]. In terms of teaching CT, this raises the question of how exactly its promotion is to be implemented in the curriculum. According to the above two uncontroversial assumptions, it may be useful to address CT in as many domains as possible and to explicitly emphasize as many intra- and interdisciplinary cross-references as possible in order to facilitate the transfer of CT principles [39,41]. However, especially in the case of scientific subjects, there are also very practical reasons to make greater use of them as a framework for promoting CT skills. First, many of our current societal problems have a large scientific component, and second, because of the close relationship between (higher-order) CT and (more domain-specific) SR, scientific subjects can be seen as an optimal framework for fostering both skills simultaneously [5,8,15,41,42,43]. Accordingly, aspects of CT are nowadays mentioned in many science education standards (e.g., [21,22,44]), but also, for example, in the PISA Science framework of the 2024 survey [7].

Despite CT’s importance, however, little robust empirical evidence or even reviews of learners’ skills levels exist. As early as 2000, Pithers and Soden criticized a lack of published research on the development of CT skills over the course of graduate programs. The research they reviewed failed to show significant positive developments in CT across different educational levels [45]. Arum and Roksa drew the same conclusion in their large-scale study among 2300 college students in the U.S. [46]. Likewise, a review by Flores et al. certified “deficient critical thinking skills” (p. 212) for U.S. college graduates [47]. Although a recent meta-analysis by Huber and Kuncel showed that students *do* develop CT skills over the course of their college years, the authors also critically noted that higher education has become less effective in promoting CT [48].

A closer look specifically at pre-service teachers reveals a picture that is almost equally concerning. In a study among 50 Indonesian pre-service teachers, Fikriyati et al. found that these teachers’ CT skills were to be classified as low [49]. The same conclusion was reached in a study among 234 Nigerian pre-service teachers by Said et al. [50]. Furthermore, Zhou et al. rated the CT skills of 69 in-service and 61 pre-service chemistry teachers as even “very low” [51] (p. 2). In line with this, in her study among 29 Saudi pre-service teachers, Gashan found that these teachers had both insufficient knowledge about CT skills and reported significant concerns about their ability to successfully encourage their future students to think critically in the classroom [52].

Taken together, all these findings suggest that the development of CT skills in school and college students needs to be carefully monitored in order to educate future citizens equipped with adequate 21st century skills. At least in the case of CT, it may be worth considering more effective support in some places.

### 1.2. Beliefs about the Nature of Science

Like well-developed CT skills, informed NOS beliefs are considered a key component of scientific literacy [8,53], which in turn should enable individuals to make evidence-based decisions about science-related personal and societal issues [54]. In a broader sense, NOS belongs to the fields of epistemology and the sociology of science [55]. According to Lederman et al., the construct refers to “science as a way of knowing, or the values and beliefs inherent to scientific knowledge and its development” [56] (p. 498; see also [57,58]).

Since the mid-20th century, NOS has received increasing attention in educational policy discourses and in science education research [57]. Over time, numerous research activities in different countries, each with their own educational systems, have led to a variety of NOS definitions [59], differing primarily in terms of which characteristics of NOS they refer to [60]. For example, some authors attach particular importance to historical or philosophical aspects of NOS (e.g., [61,62]), whereas others explicitly distinguish a “nature of scientific knowledge” (NOSK) from a “nature of scientific inquiry” (NOSI), although they also acknowledge that these two constructs are very closely intertwined (e.g., [56,63]). However, such theoretical issues seem to be more relevant for research and especially for test construction, whereas they are probably of less importance to science teaching in the classroom, as critically noted by Neumann and Kremer with respect to the NOSK–NOSI debate [64]. It is precisely this practical focus on teaching and learning that has helped to build a broad consensus among researchers in different fields, science teachers, and policymakers in science education. This consensus view contrasts with the widespread misconception that all researchers use the same kind of hypothesis-testing method that always leads to accurate and reproducible findings [65] (for an overview of common misconceptions about science, see [61]). Rather, it emphasizes that there is neither a single all-purpose scientific method nor universally valid and unshakable results. Scientific findings (including those of this study) are always tentative, socially and culturally embedded, and influenced by the researcher’s imagination, creativity, prior knowledge, methodological preferences, and, possibly, personal biases. Moreover, theories must not be equated with laws, just as observations must not be equated with conclusions [56,59,60].

In recent years, however, there have also been critical voices toward this consensus view, complaining in particular about a too-narrow conception of NOS as well as an insufficient systematization of its aspects. For example, in the context of his “Whole Science” idea, Allchin argued that aspects such as public communication about scientific views, developments, and findings should be explicitly included as part of NOS [66,67], whereas other researchers have applied the Wittgensteinian concept of family resemblance to NOS to reach a more comprehensive classification of its aspects [68,69,70]. However, even within these conceptual controversies, there is no disagreement about the fact that a sound understanding of what science and scientific inquiry are and on which principles they rely enables individuals to better understand and interpret scientific findings and to distinguish scientific claims from evidence as well as information from misinformation [5,71].

Many people, however, may have less informed NOS beliefs (e.g., [72]), perhaps exemplified by the popularity of anti-vaccine protests during the COVID-19 pandemic or, for example, rejection of evolution or denial of anthropogenic climate change [73,74,75]. In the introductory section of this article, we referred to the findings of the study by Lederman et al. [20] indicating that seventh graders in several countries have a very poor understanding of SI. However, comparable findings also exist for the public (e.g., [72,76,77]), for college students (e.g., [78,79,80,81]), and even for (pre-service) science teachers (e.g., [82,83,84,85,86,87]).

In terms of comparing college students of different majors, several studies surprisingly found either no better [80,83,88] or even worse understanding of NOS [79,89] among those of scientific versus those of non-scientific majors. Liu and Tsai also considered college students of science education majors in addition to the two groups just mentioned, but this group performed the worst of all [89]. These findings seem counterintuitive at first glance, because one would expect a better understanding of NOS particularly for college students of scientific (or science education) majors. However, several authors agree that non-scientific majors may provide better learning opportunities for the development of accurate NOS beliefs, which could be a plausible explanation for the less informed NOS beliefs of college students majoring in science [79,80,83,89].

When looking specifically at pre-service teachers, the importance of learning opportunities was also highlighted by Bruckermann et al. [90]. In their study among 232 pre-service biology teachers at 20 German universities, they found an improved understanding of NOS with increasing number of completed semesters of university teacher education. In addition, they found that pre-service teachers who were aspiring to a teaching qualification in two scientific subjects had more informed NOS beliefs than those who were aspiring to a teaching qualification in one scientific plus one non-scientific subject [90]. The latter finding may initially seem surprising in light of the reported findings on less informed NOS beliefs of college students of scientific majors [79,80,83,89]. However, it should be noted that these findings were primarily collected in the context of single-subject specialist programs, whereas the study by Bruckermann et al. focused specifically on teacher-education programs, which differ in many respects. In particular, college students in specialist programs acquire in-depth knowledge in only one specific area, whereas pre-service teachers in Germany are educated in two teaching subjects and pedagogy during the same period of time. Given this crammed teacher education curriculum, it might therefore be more advantageous to choose two teaching subjects that are related to each other (i.e., either two STEM or two HASS disciplines). Indeed, under these circumstances, learning opportunities for those aspects that overlap between the two teaching subjects chosen (such as NOS in the case of two scientific subjects) could be overall increased, despite any time constraints.

Regarding the practical design of NOS-related learning opportunities in school and college education, some methods have proven to be particularly effective: an explicit and reflective approach [91,92], problem-based learning [93], the use of SSI [65], and conceptual change as well as experiential learning [94]. According to Yacoubian [9], CT provides a helpful framework for discussions on controversial NOS issues. Furthermore, Janssen et al. presented an approach to promote a deeper understanding of NOS by integrating domain-specific and cross-domain aspects [95].

However, perhaps most importantly, empirical findings have consistently pointed to the positive effects of explicitly embedding NOS in the curriculum [18]. In addition, a study by Herman and Clough showed that purposeful implementation of comprehensive NOS courses for pre-service teachers has long-term effects that persist beyond the completion of university teacher education [53].

Considering all this knowledge about effective tools to promote an accurate understanding of NOS, the findings about naïve NOS beliefs among students and teachers, ineffective instructional practices, and inadequate curricular embedding that have been reported over and over again and that were recently reconfirmed by the Lederman et al. study [20], are of even greater concern. Thus, given the importance of informed NOS beliefs in making evidence-based decisions about science-related personal and societal issues [54], further efforts at various levels seem necessary to make science education work more effectively.

### 1.3. Research Questions and Hypotheses

The international discourse on education identifies both CT skills and NOS beliefs as important variables whose curricular embedding needs to be substantiated and emphasized more explicitly than in the past. Skills in the domains of both constructs are teachable and learnable, although, as previous findings (e.g., [20]) point out, often not learned well. To be effective in promoting certain competencies, teachers themselves must have a high level of expertise in the respective domains. Against this background, we wanted to address the following research questions (RQ) and hypotheses (H):

- RQ1: How do pre-service biology teachers perform on tests that assess their CT skills and their NOS beliefs? Given the findings mentioned in the above sections (e.g., [11,50,84,85]), we expected their CT skills to be average at best (H1) and their NOS beliefs to not reach an informed level (H2).
- RQ2: Does test performance differ depending on the number of learning opportunities? Because previous findings pointed to the importance of learning opportunities for the development of CT skills (e.g., [41]) and NOS beliefs (e.g., [90]), we expected pre-service biology teachers enrolled in the master’s program to perform better on both variables than those enrolled in the bachelor’s program (H3). In addition, we expected those who were aspiring to a teaching qualification in two scientific subjects to outperform those who were aspiring to a teaching qualification in only one scientific plus one non-scientific subject (H4). The latter assumption seems plausible considering an overall increase in specific learning opportunities for NOS [90], which in turn could also have a positive impact on CT skill development [43,96].
- RQ3: What is the relationship between CT skills and NOS beliefs? Does it differ between the groups compared to answer RQ2? The available literature suggests that both NOS frameworks are useful for developing CT skills [8,97] and, conversely, CT frameworks are useful for developing NOS beliefs [9,98]. Given this, we expected a positive relationship between both constructs (H5). Considering a different number of learning opportunities (see H3 and H4), we further expected this relationship to be closer for pre-service biology teachers enrolled in the master’s program than for those enrolled in the bachelor’s program (H6), and likewise to be closer for those who were aspiring to a teaching qualification in two scientific subjects than for those who were aspiring to a teaching qualification in only one scientific plus one non-scientific subject (H7).

## 2. Materials and Methods

In most German schools, “science” is not taught as one single subject but in separate chemistry, biology, and physics lessons. Accordingly, during university teacher education, most pre-service science teachers are prepared to teach only a single scientific discipline. However, answering our RQ separately for all groups of pre-service teachers of each scientific discipline would not have been reasonable from a test-economics point of view, since the research background (especially in Germany) is rather weak. Therefore, we decided to answer our RQ exclusively for a sample of pre-service biology teachers at this stage. The discipline of biology was chosen because, especially in light of current global challenges (climate change, biodiversity loss, COVID-19 pandemic, etc.), the fundamental importance of biological knowledge for our modern world is becoming more and more evident (e.g., [3]). Therefore, in any case, biology teachers are expected to have a broad expertise in the domains of CT and NOS in order to effectively promote these competencies in the biology classroom [27].

In the spring of 2022, we conducted a cross-sectional assessment at one German university. Participants were invited in the context of university courses on biology education given by colleagues known to us. A total of *n* = 151 pre-service teachers took part in our study, of which *n* = 27 were aspiring to a teaching qualification in two scientific subjects whereas the *n* = 124 remaining participants were aspiring to a teaching qualification in biology plus one subject belonging to humanities, arts, or social sciences. Approximately 77% of the sample were female and 23% were male. On average, the participants were 23.54 (*SD* = 3.45) years old and had already completed slightly less than 3 years of their 5-year university teacher education. In total, *n* = 73 of them were enrolled in a bachelor’s program, *n* = 76 were enrolled in a master’s program, and *n* = 2 did not provide information about their degree program.

### 2.1. Assessments

We asked the participants to complete an online assessment covering both their CT skills and their NOS beliefs. For this purpose, we used the Qualtrics Survey software (SAP America, Newtown Township, PA, USA) [99]. Data collection took place in the presence of a test administrator during the teaching time of the university courses selected.

#### 2.1.1. Critical Thinking Skills

To assess our participants’ CT skills, we used the Watson–Glaser Critical Thinking Appraisal (WGCTA) [100,101,102]. It is the oldest and probably best-known standardized test for the assessment of decontextualized CT skills [103] and is the only one available in an official German-language adaptation. The WGCTA asks participants to complete 56 single-choice items of varying difficulty, covering a total of five generic CT skills (Table 1).

Since the WGCTA belongs to the category of power tests, there is no time limit for its completion (typically, the required time is 30–40 min). Notwithstanding convincing validity evidence (e.g., [104,105]), issues have been identified regarding the reliability of the subscales and the factorial structure of the WGCTA [103]. Because a one-factor solution currently appears to be safest, we therefore considered only the total WGCTA score in our study. For our sample, the homogeneity was α_KR-20_ = 0.66.

#### 2.1.2. Beliefs about the Nature of Science

Our participants’ NOS beliefs were assessed using Liang et al.’s Student Understanding of Science and Scientific Inquiry (SUSSI) test, which was developed specifically for targeting pre-service teachers [106]. Overall, six NOS aspects are covered by the SUSSI (Table 2).

The participants were asked to rate a total of 24 Likert-type items according to how much they agreed with them (1 = *strongly disagree* to 5 = *strongly agree*), with higher agreement reflecting more accurate NOS beliefs (after recoding negative items). When analyzing the data, it is possible not only to calculate sum scores but also to classify the responses. For this purpose, the four responses per NOS aspect are analyzed according to whether none (= naïve) or all four (= informed) of them received a score > 3; the range in between corresponds to transitional beliefs [106]. Subsequently, of course, aggregation can be used to determine the overall accuracy of NOS beliefs. The SUSSI’s additional qualitative part, consisting of 12 open-ended items, was not used in our study to make the survey as time-efficient and motivating as possible for our participants. This approach is in line with a suggestion by Miller et al., who found substantial correlations between the Likert-type and open-response items [80]. Despite convincing evidence for validity [106], low internal consistencies of single subscales have been found [107]. Therefore, we decided to calculate only the reliability of the total scale. Apart from that, we consider the low internal consistencies of the subscales an indication that there might be problems with the factorial structure [108]. Therefore, to be on the safe side, we decided to consider only the total score, just as in the case of the WGCTA. For our sample, the homogeneity was α_Cronbach_ = 0.70.

#### 2.1.3. Need for Cognitive Closure

NCC was assessed as a potential covariate using a short scale by Schlink and Walther [109]. This short scale is based on the NCC scale by Webster and Kruglanski, a 42-item scale covering the five NCC facets (1) preference for order, (2) preference for predictability, (3) decisiveness, (4) discomfort with ambiguity, and (5) closed-mindedness [33]. As explained in Section 1.1, the NCC construct represents just the opposite of important CT dispositions [31], which in turn may facilitate the application of CT skills [32]. Conversely, then, high levels of NCC can be expected to be a barrier to CT [34,35,36,37,38]. However, because dispositions are assumed to be rather stable [110,111], it seems unreasonable to expect them to be as changeable through academic training as (more variable) skills. Therefore, consistent with the findings of Rosman et al. [112], we decided not to consider NCC as a dependent variable but to test for baseline differences on this construct before comparing any groups in our study. Schlink and Walther’s short scale consists of 16 items and has demonstrated good psychometric properties in validation studies [109]. An example item is “Looking at a problem from different perspectives only leads to confusion” [109] (p. 156). For our sample, the homogeneity was α_Cronbach_ = 0.75.

### 2.2. Statistical Methods

RQ1 was answered on a descriptive level only. In the case of the WGCTA, we used the standard scores provided in the test manual to determine *T* scores for each participant. This type of standard score has a mean of 50 and a standard deviation of 10. When interpreting *T* scores, a respondent’s test result is compared with results of a reference group. A *T* score of 50 corresponds to an average score for the reference group, whereas a *T* score of 60, for example, indicates a test result being one standard deviation above the mean. In general, *T* scores above 60 or below 40 are considered “clinically” significant by indicating a potential problem or issue that may require further action [113]. The reference group chosen in our study was intended to be as similar as possible to our sample. Therefore, we selected the “young professionals” group, consisting of *n* = 108 university students as well as young people from Germany who had just entered professional life after graduating from university. In the case of the SUSSI, we followed the recommendations of Liang et al. [106] by categorizing the individual response patterns of our participants into naïve, transitional, and informed NOS beliefs.

To answer RQ2, we first tested for relevant baseline differences between the compared groups to decide whether covariates or moderator variables should be included in further analysis. Potential differences on categorical variables were checked using *χ*^2^ tests, whereas differences on metric variables were checked using *t*-tests or Mann–Whitney *U* tests (if there was no group-wise normal distribution). After finding no relevant baseline differences (see Section 3.2), the same statistical procedures were retained for the analysis of differences on the dependent variables.

Regarding the role of learning opportunities, the comparison of participants of the bachelor’s program (BA group) with those of the master’s program (MA group) was comparatively easy to carry out due to almost equal group sizes. However, in examining the influence of teaching subject-related learning opportunities, we faced the problem of drastically unequal-sized groups, as only *n* = 27 out of *n* = 151 participants were aspiring to a teaching qualification in two scientific subjects (2S group). Because comparing such unequal-sized groups is not unproblematic in terms of type I errors and statistical power [114,115], we took a random sample of *n* = 27 out of the *n* = 124 remaining participants who were aspiring to a teaching qualification in only one scientific plus one non-scientific subject (1S group). Statistical comparability of these two groups with respect to potential covariates was ensured afterward.

RQ3 was answered by calculating Spearman correlations between the participants’ WGCTA and SUSSI scores, both for the total sample and for the groups compared to answer RQ2. Subsequently, a Fisher *z*-transformation was carried out to test whether the correlations found were significantly different between the BA vs. MA and the 1S vs. 2S groups, respectively. Although this method is actually intended for comparing two Pearson correlation coefficients, simulation studies have shown that it can also be used for comparing rank correlations and, moreover, that it is preferable to alternative methods due to a lower risk of type I errors [116].

## 3. Results

In the following, we present the results of our statistical analyses separately for each RQ. An overview of relevant baseline characteristics of our sample can be found within the results report on RQ2.

### 3.1. RQ1: How Do Pre-Service Biology Teachers Perform on Tests That Assess Their CT Skills and Their NOS Beliefs?

**Figure 1:**
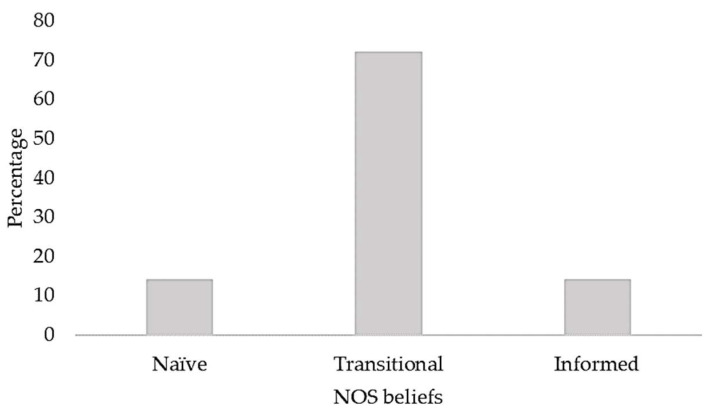
Distribution of the participants’ NOS beliefs according to their respective SUSSI score.

Regarding their CT skills, our participants achieved an average WGTCA *T* score of 42.42 (*SD* = 8.43), which is just within the average range (from 40 to 60). As for NOS beliefs, our participants achieved an average sum score of 79.42 (*SD* = 9.66), which in principle can vary from 24 (minimum) to 120 (maximum). Accordingly, the response patterns of a vast majority (72%) can be classified as transitional beliefs (Figure 1).

### 3.2. RQ2: Does Test Performance Differ Depending on the Number of Learning Opportunities?

Table 3 provides an overview of descriptive parameters and the preliminary analysis results regarding potential baseline differences. Overall, there was a negative, albeit weak, association between CT skills and NCC (*r* = −0.23, *p* < 0.01), which was expected. In both the two degree program-related groups (BA and MA) and the two teaching subject-related groups (1S and 2S) the respective gender distributions were comparable. Likewise, the proportions of students who were aspiring to a teaching qualification in only one vs. two scientific subjects in the BA and MA groups (1S/2S ∪ BA/MA) as well as the proportions of participants enrolled in a bachelor’s vs. a master’s program in the 1S and 2S groups (BA/MA ∪ 1S/2S) were comparable. On the metric variables, only the BA and MA groups showed significant differences regarding their age and semester of study, as expected by definition, but they did not differ significantly in terms of their NCC. The 1S and 2S groups did not show any significant differences on the metric variables. Given this, none of these variables needed to be included as a covariate or moderator variable in further analyses.

The results of inferential testing regarding CT skills and NOS beliefs of the compared groups are shown in Table 4. Although the MA group scored significantly higher than the BA group regarding their CT skills (*d* = 0.46), the two groups were found to be comparable in terms of their NOS beliefs. For the 1S and 2S groups, significant differences were found on both variables in favor of the 2S group (*d* = 0.71 for both CT skills and NOS beliefs).

### 3.3. RQ3: What Is the Relationship between CT Skills and NOS Beliefs? Does It Differ between the Groups Compared to Answer RQ2?

Among the total sample, there was a significant medium correlation between CT skills and NOS beliefs. The same result was found among the BA and MA groups. Although the relationship was closer for the MA group than for the BA group, the difference was not found to be statistically significant. Among the 1S and 2S groups, a significant correlation was found only for the 2S group. Although this correlation corresponded to a large effect, it again did not result in a statistically significant group difference in terms of correlation levels (Table 5).

## 4. Discussion

In our study, we investigated how pre-service biology teachers from Germany perform on tasks assessing their CT skills and NOS beliefs (RQ1), and whether test performance (RQ2) and/or the relationship between CT skills and NOS beliefs (RQ3) differ as a function of the number of learning opportunities.

To this end, we conducted a cross-sectional study including *n* = 151 participants. Their CT skills were assessed using the WGCTA [100,101,102] and their NOS beliefs were assessed using the SUSSI test [106]. To answer RQ1, we used standard *T* scores provided in the test manual in the case of the WGCTA; for the SUSSI test, we followed the recommendations of Liang et al. [106] by categorizing the participants’ test scores into (a) naïve, (b) transitional, and (c) informed NOS beliefs. To answer RQ2, we ran group comparisons using *t*-tests and Mann–Whitney *U* tests. This involved operationalizing the number of learning opportunities by both degree program (BA group vs. MA group) and by subjects for which a teaching qualification was aspired to (1S group vs. 2S group). RQ3 was answered by calculating Spearman correlations between the participants’ WGCTA and SUSSI scores. Subsequently, a Fisher *z*-transformation was done to test whether the correlations found were significantly different between the BA vs. MA and the 1S vs. 2S groups, respectively.

Our results show that the CT skills of the pre-service biology teachers sampled were only in the low–average range and that 86% of them did not have an informed understanding of NOS. However, we also found a significant positive effect of the number of learning opportunities, albeit in the case of NOS beliefs, that effect was less related to the degree program. A consistent benefit was instead found for pre-service teachers who were aspiring to a teaching qualification in two scientific subjects. In addition, a higher number of learning opportunities (irrespective of whether degree program- or teaching subject related) generally tended to result in a closer association between CT skills and NOS beliefs.

### 4.1. RQ1: How Do Pre-Service Biology Teachers Perform on Tests That Assess Their CT Skills and Their NOS Beliefs?

Based on several findings suggesting that the CT skills and the understanding of NOS of different populations are in need of improvement, we assumed that our participants’ CT skills would be average at best (H1) and that their NOS beliefs would not reach an informed level (H2). Compared to the WGCTA norm sample of young professionals, our sample of pre-service biology teachers achieved a test score that (just barely) corresponds to average CT skills. Thus, H1 was supported by the results. In terms of their NOS beliefs, only 14% of our participants achieved a SUSSI score corresponding to an informed understanding, whereas the remaining participants achieved scores indicating a transitional (72%) or even a naïve level (14%). Thus, H2 was also supported by the results.

CT is an important 21st century skill that various researchers, educational policymakers, and economists consider to be central in coping with future societal problems [6]. Although school is *the* place where CT skills can be acquired, empirical evidence from educational research on students’ and (pre-service) teachers’ CT skills is quite limited compared to, for example, that on NOS beliefs. Therefore, we hope that our study has made a valuable contribution to strengthening this knowledge base. Our finding of only low–average CT skills among the pre-service biology teachers in our sample implies some potential for re-evaluating university teacher education programs in this regard. Teachers play a key role in promoting CT among their students. Science education in particular provides an excellent setting for encouraging students to think critically, as the two constructs of CT and SR are closely linked. However, effective teaching can only be successful if science teachers themselves are sufficiently skilled in CT [27]. Such expertise, in turn, can only be developed if pre-service science teachers are given adequate opportunities to acquire CT skills and, more importantly, to practice how to teach them most effectively in the classroom [117] (see Section 4.2). However, whether such a curricular framework for university science teacher education already exists everywhere remains highly questionable in light of Lederman et al.’s [20] comments.

Regarding the finding of less-informed NOS beliefs among the participants of our sample, there is not much left to say, as our results simply paint the same picture as numerous previous studies on pre-service science teachers’ understanding of NOS (see Section 1.2). Nevertheless, one could argue that our participants still had, on average, about two years of university teacher education to complete, during which they might develop NOS beliefs that will correspond to an informed level in the end. However, Bruckermann et al. argued that NOS-related learning opportunities tend to be concentrated in the first semesters of the bachelor’s program, with their number stagnating thereafter (especially in the master’s program) [90]. That some of this stagnation may be directly reflected in the development of NOS beliefs was suggested by Mahler et al.’s longitudinal study on the development of NOS beliefs over the course of university teacher education [118], which, like Bruckermann et al.’s study, focused on pre-service teachers in Germany. Thus, the hope for a significantly positive further development of our participants’ NOS beliefs until the completion of their university teacher education can at least be questioned.

### 4.2. RQ2: Does Test Performance Differ Depending on the Number of Learning Opportunities?

Based on previous findings that pointed to the importance of learning opportunities for the development of CT skills and NOS beliefs, we assumed that the MA group would perform better on both variables than the BA group (H3). In addition, we expected that pre-service teachers who were aspiring to a teaching qualification in two scientific subjects would outperform those who were aspiring to a teaching qualification in only one scientific plus one non-scientific subject (H4). In terms of the degree program, we found a significantly better test performance of the MA group regarding their CT skills, which corresponded to a medium effect size. However, both the BA and the MA group performed comparably with respect to their NOS beliefs. Thus, H3 was only partially supported by the results. Regarding teaching subject-related differences, we found significantly better test performances in the 2S group in terms of both their CT skills and their NOS beliefs, which corresponded to medium effect sizes in each case. Thus, H4 was supported by the results.

As discussed with respect to RQ1, the fact that the BA and MA groups did not show significantly different NOS understandings is consistent with Bruckermann et al.’s explanations regarding the stagnation of the number of NOS-related learning opportunities after the first semesters of university teacher education [90]. With this in mind, however, the significant differences between the BA and MA groups in terms of their CT skills conversely suggest that CT-related learning opportunities might be more continuously spread across the years of study. The finding of significantly better CT skills among participants of the MA group is therefore consistent with the results of Huber and Kuncel’s meta-analysis, which showed, in contrast to previous studies, that the college years are indeed beneficial for the development of students’ CT skills [48].

In addition, the significantly better test performance of the 2S group compared to the 1S group suggests that the choice of two scientific teaching subjects might positively affect the development of both CT skills and NOS beliefs of pre-service teachers, regardless of whether they are enrolled in a bachelor’s or a master’s program. This result is again aligned with the findings of Bruckermann et al. [90]. Overall, there are several possible explanations for the 2S group’s better test performance: On the one hand, the 2S group participants may in total have had more learning opportunities to acquire CT skills and/or develop NOS beliefs. Whether the “and” or the “or” is true cannot be determined on the basis of our results. Since there appears to be an important relationship between the two variables (see Section 3.3), it may well be possible that more learning opportunities that are specific to one of the two constructs lead to positive developments of the related one. Likewise, it remains an open question whether learning opportunities merely have an additive effect or whether the whole is more than the sum of its parts in the end. Based on Hartmann et al.’s work on SR skills, there appears to be an additional positive effect of learning opportunities across different content domains [119]. Considering the close relationship between the constructs of SR, CT, and NOS (see Section 1), it seems reasonable to assume a comparable effect regarding the development of CT skills and NOS beliefs. On the other hand, it may also be possible that other factors that were not considered in this study and that may be only indirectly related to the choice of teaching subjects are (partly) responsible for the differences observed between the 1S and 2S groups (see Section 4.4). However, which of these explanations is or are true cannot be determined within the scope of this study. Future studies with appropriate longitudinal or cross-lagged panel designs would be needed for this purpose.

### 4.3. RQ3: What Is the Relationship between CT Skills and NOS Beliefs? Does it Differ between the Groups Compared to Answer RQ2?

Given reasoned suggestions in the literature that CT frameworks may be useful for developing NOS beliefs and vice versa, we expected a positive relationship between the two constructs (H5). Furthermore, we hypothesized that this relationship would be closer for the MA group than for the BA group (H6), and likewise, that it would be closer for 2S group than for the 1S group (H7). Regarding the relationship between CT skills and NOS beliefs, we found a significant positive correlation among the total sample, corresponding to a medium effect. Thus, H5 was supported by the results. Similarly, there was a significant medium correlation between the two variables among both the BA and MA groups. Despite the fact that the relationship was nominally closer for the MA group, the difference did not prove to be statistically significant. H6 was therefore supported only at a nominal and not at a statistical level. Between the 1S and 2S groups, a significant correlation was found only for the 2S group. However, there was also no statistically significant group difference in terms of correlation levels. H7 was therefore also supported only at a nominal and not at a statistical level.

The medium correlation between CT skills and NOS beliefs implies some closeness of the constructs in terms of required skills, suggesting that CT- and NOS-based instructional settings could be used effectively for reciprocal promotion (see Section 1.3).

Furthermore, our results show that among both the MA (compared to BA) and 2S groups (compared to 1S), the two constructs tended to be more closely related. We attribute the fact that neither the correlation between CT skills and NOS beliefs among participants in the 1S group nor the differences in correlation levels in the two group comparisons of BA vs. MA (*p* = 0.184) and 1S vs. 2S (*p* = 0.093) proved statistically significant to the comparatively small group sizes (see Section 4.4). However, regardless of the question of statistical significance, it should be noted that, particularly for pre-service teachers who were aspiring to a teaching qualification in two scientific subjects, the test performances on both constructs became more consistent. Considering the results found for RQ2, this convergence in scoring is likely the result of a consistently better understanding of NOS, which in turn could once again be related to an increased number of learning opportunities offered to the 2S group participants.

One resulting practical implication is that the closer the relationship, the more efficiently a CT framework may be used to promote accurate NOS beliefs and the more efficiently an NOS framework may be used to promote CT. This means that, especially for pre-service teachers who are aspiring to a teaching qualification in two scientific subjects, there are better options for doing so, regardless of the degree program. Conversely, this also means that for those pre-service teachers who are aspiring to a teaching qualification in only one scientific plus one non-scientific subject, the implementation of more learning opportunities or of courses dealing with content and perspectives from other scientific disciplines (see Section 4.2), respectively, might lead to better developed CT skills and a more informed understanding of NOS (as well as a closer relationship between both constructs).

### 4.4. Limitations

Although our study yielded interesting and valuable findings, it is important to evaluate them in light of some limitations:

1. All of the pre-service teachers in our sample were studying at only one German university, which undoubtedly limits the generalizability of our results. It seems essential to conduct studies with a comparable focus in other contexts (e.g., other universities, other countries) for a broader and more valid insight.
2. For reasons of test economy, we decided to answer our RQ exclusively for pre-service biology teachers at this stage. Therefore, it remains uncertain whether similar results may also be found for groups of pre-service chemistry or physics teachers. Future studies focusing on this specific question could provide valuable information on whether there are comparable or differential effects.
3. In terms of our study’s internal validity, it should be noted that possible self-selection effects might have contributed to the differences found between the 1S and 2S groups. For example, it seems possible that 2S group participants already had a stronger interest in science, and thus perhaps a better understanding of NOS, prior to entering their teacher education program. Furthermore, such potential baseline differences could have been intensified by major-specific communication and interaction (e.g., [120]). To validly estimate such mediating and moderating effects, future research would require longitudinal designs and appropriate baseline measures at the beginning of university teacher education.
4. In the case of the RQ3-related statistical analyses, an adequate level of power was not achieved in some cases, resulting in a quite high risk of type II error [121]. Regarding the correlation analysis for the 1S group, the power was only 0.52, implying that 55 instead of 27 participants would have been needed to detect the medium effect of *r*_CT―NOS_ = 0.33 with sufficient power of 0.80. This problem did not exist in the case of the 2S group, where the power was sufficient to detect the large effect of *r*_CT―NOS_ = 0.62. In a comparable manner, the power of the two group comparisons in terms of correlation levels was only 0.55 (BA vs. MA) and 0.27 (1S vs. 2S), respectively. Both comparisons would have required group sizes of 141 participants each to detect medium effects with sufficient power of 0.80. Therefore, a replication of our study that takes a larger sample in order to clearly prove all effects at an adequate level of statistical power would be desirable.

## 5. Conclusions

Despite the limitations discussed above, our study provides interesting ideas for future research and practice. Although our findings on less informed NOS beliefs of pre-service biology teachers simply add to a long-known picture, our findings on CT skills, which were also rather poorly developed in our sample, hopefully do much more to strengthen the evidence base in this regard. Most importantly, to the best of our knowledge, our study is the first one that simultaneously considers both variables, CT skills and NOS beliefs of pre-service science teachers. On the one hand, our results suggest that learning opportunities during university teacher education might play a crucial role in the development of these competencies. The finding of comparable NOS beliefs among participants in both the BA and the MA group is consistent with the assumption of some stagnation in the number of NOS-related learning opportunities after the first semesters of university teacher education [90]. With this in mind, however, the significant group differences in terms of CT skills conversely indicate that CT-related learning opportunities might be more continuously spread across the course of study. Furthermore, the significantly better test performance of the 2S compared to the 1S group suggests that the choice of two scientific (compared to only one scientific plus one non-scientific) teaching subjects seems to be particularly beneficial. On the other hand, our correlational findings provide empirical evidence of the potential effectiveness of CT- and NOS-based instructional settings proposed in the literature for promoting the respective other skill [8,9,97,98].

With regard to future research, interesting questions can be inferred from our results, especially with respect to the influence of learning opportunities. One of these questions, for instance, concerns the extent to which implementing learning opportunities that address the content and perspectives of other scientific disciplines might provide a particular advantage in university biology, chemistry, or physics teacher education. If such an advantage was shown, it would be advisable to offer more interdisciplinary learning opportunities in science teacher education.

Finally, if our findings were replicated in other contexts, it would be worth considering how pre-service science teachers’ CT skills and NOS beliefs might be better fostered during university teacher education. Given the interrelationship between the two constructs, it might be useful to adopt instructional approaches such as the discussion of controversial SSI to simultaneously promote CT skills and NOS beliefs [6] and possibly even have a mutually beneficial effect on their development.

To summarize, being a teacher is linked to the invaluable potential to promote competencies of several generations of future citizens and thus to make an important contribution to solving future societal issues. However, if teachers limit their role to merely transmitting subject-matter knowledge, they are likely to limit their students’ competencies to their own knowledge base. In other words, teachers must enable their students to develop competencies beyond that. Science teachers can do this, for example, by engaging their students in thinking critically and helping them to develop an appropriate understanding of NOS. These days, an extensive knowledge base from empirical research makes it more possible than ever for teachers to improve the teaching and learning of CT and NOS in the classroom. However, it has to be acknowledged that students’ competencies are highly dependent on their teachers’ competencies [122]. Therefore, teachers themselves must be qualified experts in the domains of CT and NOS. This, in turn, requires university teacher education programs that provide pre-service teachers with opportunities to acquire expertise in relevant domains and to practice applying and, more importantly, teaching related skills in the classroom. To achieve this goal, further efforts seem necessary in many places. On the one hand, such efforts certainly concern political fields of action. On the other hand, however, the competencies of university educators might also be addressed, since the competencies of pre-service teachers are, of course, in the same way dependent on the expertise of *their* teachers.

## Figures and Tables

**Table 1:** CT skills covered by the WGCTA.

CT Skill	Ability to …
Inference (19 items)	… rate the probability of truth of inferences based on given information
Recognition of assumptions (8 items)	… identify unstated assumptions or presuppositions underlying given statements
Deduction (12 items)	… determine whether conclusions follow logically from given information
Interpretation (8 items)	… weigh evidence and decide whether data-based generalizations or conclusions are justifiable
Evaluation of arguments (9 items)	… evaluate the strength and relevance of arguments regarding a particular issue

**Table 2:** NOS aspects covered by the SUSSI test.

NOS Aspect	Related (Informed) Views
Observations and inferences (4 items)	Science is based on both observations (descriptive statements) and conclusions (interpretations of observations), which in turn are guided by current and diverse scientific perspectives.
Tentativeness (4 items)	It is reasonable to have confidence in scientific findings, but one should be aware that these findings are subject to change and may be revised considering new evidence.
Scientific theories and laws (4 items)	Scientific theories (well-substantiated explanations of some aspect of the natural world) explain (some) scientific laws (generalized relationships of natural phenomena under certain conditions) but are clearly distinguishable from them.
Social and cultural embeddedness (4 items)	As a human endeavor, scientific practice as well as interpretations and acceptance of scientific results are influenced by the particular reference society and culture.
Creativity and imagination (4 items)	Scientists use their imagination and creativity throughout their scientific investigations (e.g., in generating hypotheses and theories or in explaining scientific results).
Scientific methods (4 items)	Different scientific disciplines use different methods, theories, and standards to generate scientific knowledge, so there is no one-size-fits-all scientific approach that all scientists follow.

**Table 3:** Results of preliminary analyses regarding potential baseline differences.

Baseline Characteristics		Total Sample	BA	MA	1S	2S
		*n* = 151	*n* = 73	*n* = 76	*n* = 27	*n* = 27
Gender		77% female 23% male	75% female 23% male 2% other	78% female 22% male	70% female 30% male	70% female 30% male
1S/2S ∪ BA/MA and BA/MA ∪ 1S/2S		n/a ^1^	84% 1SM 16% 2SM	80% 1SM 20% 2SM	54% BA 46% MA	56% BA 44% MA
Age	*M*	23.54	22.25	24.82	24.30	23.07
	*SD*	3.45	2.53	3.80	3.94	3.19
Semester	*M*	5.93	4.49	7.30	5.85	6.33
	*SD*	1.63	1.06	0.52	1.43	1.44
NCC	*M*	3.37	3.40	3.37	3.25	3.27
	*SD*	0.61	0.57	0.63	0.78	0.54
**Difference Testing**	Gender		$\chi^{2}$ = 1.08, *p* = 0.583		$\chi^{2}$ = 0.00, *p* = 1.000	
	1S/2S ∪ BA/MA and BA/MA ∪ 1S/2S		$\chi^{2}$ = 0.27, *p* = 0.601		$\chi^{2}$ = 0.47, *p* = 0.494	
	Age		*U* = 4281.50, *p* < 0.001		*U* = 295.50, *p* = 0.227	
	Semester		*U* = 5548.00, *p* < 0.001		*U* = 285.00, *p* = 0.222	
	NCC		*U* = 2453.00, *p* = 0.248		*t*(50) = 0.10, *p* = 0.460	

^1^ Not applicable.

**Table 4:** Results of difference-testing analyses regarding CT skills and NOS beliefs.

Group	*n*	WGCTA		SUSSI		Difference Testing	
		*M*	*SD*	*M*	*SD*		
BA	73	40.60	7.78	79.36	8.43	WGTCA	*U* = 3489.50, *p* < 0.01
MA	76	44.38	8.66	79.63	10.84	SUSSI	*t*(141.01) = 0.17, *p* = 0.432
1S	27	38.56	6.53	75.92	7.93	WGTCA	*U* = 506.50, *p* < 0.01
2S	27	44.11	8.87	82.74	9.01	SUSSI	*U* = 505.50, *p* < 0.01

**Table 5:** Results of correlation analyses and comparisons of relationships between CT skills and NOS beliefs.

Group	*n*	*r*_CT―NOS_ ^1^	*p*	Difference Testing
Total sample	151	0.36	<0.001	n/a ^2^
BA	73	0.30	<0.05	*z* = 0.90, *p* = 0.184
MA	76	0.43	<0.001	
1S	27	0.33	n/s ^3^	*z* = 1.32, *p* = 0.093
2S	27	0.62	<0.001	

^1^ Spearman correlation between the participants’ CT skills and NOS beliefs; ^2^ not applicable; ^3^ not significant.

## Data Availability

The data presented in this study are available on request from the corresponding author. The data are not publicly available due to legal and privacy issues.
